# Comprehensive Transcriptome Profiling Reveals Long Noncoding RNA Expression and Alternative Splicing Regulation during Fruit Development and Ripening in Kiwifruit (*Actinidia chinensis*)

**DOI:** 10.3389/fpls.2016.00335

**Published:** 2016-03-29

**Authors:** Wei Tang, Yi Zheng, Jing Dong, Jia Yu, Junyang Yue, Fangfang Liu, Xiuhong Guo, Shengxiong Huang, Michael Wisniewski, Jiaqi Sun, Xiangli Niu, Jian Ding, Jia Liu, Zhangjun Fei, Yongsheng Liu

**Affiliations:** ^1^Department of Biological Sciences, School of Biotechnology and Food Engineering, Hefei University of TechnologyHefei, China; ^2^Ministry of Education Key Laboratory for Bio-Resource and Eco-Environment, College of Life Science, State Key Laboratory of Hydraulics and Mountain River Engineering, Sichuan UniversityChengdu, China; ^3^Section of Plant Biology, Boyce Thompson Institute for Plant Research, Cornell UniversityIthaca, NY, USA; ^4^U.S. Department of Agriculture – Agricultural Research ServiceKearneysville, WV, USA; ^5^Sichuan Technical Exchange CenterChengdu, China

**Keywords:** fruit development and ripening, long noncoding RNAs, alternative splicing, novel genes, transcriptome profiling, gene expression

## Abstract

Genomic and transcriptomic data on kiwifruit (*Actinidia chinensis*) in public databases are very limited despite its nutritional and economic value. Previously, we have constructed and sequenced nine fruit RNA-Seq libraries of *A. chinensis* “Hongyang” at immature, mature, and postharvest ripening stages of fruit development, and generated over 66.2 million paired-end and 24.4 million single-end reads. From this dataset, here we have identified 7051 long noncoding RNAs (lncRNAs), 29,327 alternative splicing (AS) events and 2980 novel protein-coding genes that were not annotated in the draft genome of “Hongyang.” AS events were demonstrated in genes involved in the synthesis of nutritional metabolites in fruit, such as ascorbic acids, carotenoids, anthocyanins, and chlorophylls, and also in genes in the ethylene signaling pathway, which plays an indispensable role in fruit ripening. Additionally, transcriptome profiles and the contents of sugars, organic and main amino acids were compared between immature, mature, and postharvest ripening stages in kiwifruits. A total of 5931 differentially expressed genes were identified, including those associated with the metabolism of sugar, organic acid, and main amino acids. The data generated in this study provide a foundation for further studies of fruit development and ripening in kiwifruit, and identify candidate genes and regulatory elements that could serve as targets for improving important agronomic traits through marker assisted breeding and biotechnology.

## Introduction

The genus *Actinidia*, commonly known as kiwifruit, is widely distributed throughout most of East Asia, and consists of 57 species of climbing plants (Ferguson and Huang, 2007). This genus is considered native to China, since most of the species occur in the southwest of the country. Over the past three decades, kiwifruit has become an economically important fruit crop due to its nutritional quality and unique flavor, and its cultivation worldwide has substantially increased (Ferguson and Huang, 2007; Zhang L. et al., 2010). The annual production of kiwifruit was 3.26 million metric tons in 2013 (http://faostat3.fao.org/). Despite its importance and increasing production, the international kiwifruit industry relies mainly on a few naturally selected cultivars derived from two intraspecific taxa, *A. chinensis* and *A. deliciosa* (Chat et al., 2004; Zhang L. et al., 2010).

The flesh of the majority of kiwifruit cultivars is either green or yellow at harvest (Huang and Ferguson, 2003; Wang et al., 2003; Montefiori et al., 2005; Crowhurst et al., 2008). The first commercial red-flesh cultivar is *A. chinensis* “Hongyang.” It is characterized by a medium fruit size (average weight per fruit is 77.6 g), a total acid content of 0.49%, and a soluble solid concentration of 19.6%. Importantly, “Hongyang” also has a high content of vitamin C (136 mg 100 g^−1^ FW) and anthocyanin (2.99 mg 100 g^−1^ FW; Wang et al., 2003; Montefiori et al., 2005). Kiwifruit contains sugars such as glucose, fructose, and sucrose, and organic acids such as citric, quinic, malic, and ascorbic acids, and main amino acids such as glutamine, arginine, and aspartate (Redgwell and MacRae, 1992; Capitani et al., 2010). The combination of sugars, organic acids and free amino acids represents the major factor contributing to kiwifruit flavor (Sorrequieta et al., 2010). In general, the total sugar content in “Hongyang” is higher than that of other *A. chinensis* cultivars; while the concentration of the three main organic acids (citric acid, quinic acid and malic acid) is similar (Wang et al., 2003; Nishiyama et al., 2008). This sugar/acid ratio is believed to contribute to the excellent flavor characteristics for “Hongyang” fruit. Carotenoids, chlorophylls, and anthocyanins in kiwifruit represent forms of dietary antioxidants. Anthocyanins are the main pigments in “Hongyang” fruit inner pericarp, chlorophylls and lutein are the main pigments in outer pericarp, all of them contributing to its overall appearance and attractiveness (Montefiori et al., 2005; Nishiyama et al., 2008).

The critical genes in the flavonoid (Montefiori et al., 2011; Huang et al., 2013; Jaakola, 2013) or monoterpene (Nieuwenhuizen et al., 2015) synthesis pathway have been characterized in fruits within a range of plant species. The major anthocyanin in *A. chinensis* “Hongyang,” is cyanidin 3-O-xylo-(1-2)-galactoside, and smaller amounts of cyanidin 3-O-galactoside are present (Montefiori et al., 2005). In contrast, cyanidin 3-O-xylo(1-2)-galactoside has not been detected in *A. deliciosa* genotypes, and the major reported anthocyanins are cyanidin 3-O-galactoside and cyanidin 3-O-glucoside (Montefiori et al., 2005, 2011; Fraser et al., 2013). Anthocyanin biosynthesis and accumulation are regulated by many transcription factors, as well as environmental factors. The precise control of anthocyanin accumulation in inner pericarp tissues of kiwifruit, however, has not been elucidated. “Hongyang” with its unique flavor, excellent nutritional quality and high market value (Jaeger and Harker, 2005) represents an excellent system to study kiwifruit development and ripening.

Sweetness is one of the most important quality traits for kiwifruit cultivation and breeding. Sugars (sucrose, monosaccharides, and polyols) are important molecules in plants and function as a source of energy, building blocks for cell walls, and as osmotic and regulatory molecules (Smeekens et al., 2010). Enzymes associated with sugar metabolism include ADP-glucose pyrophosphorylase (AGPase), sucrose phosphate synthase (SPS), invertase (INV), amylase, sucrose synthase (SUS), fructokinase (FK), and hexokinase (HK; Deluc et al., 2007). Sugar transporters are essential proteins for the transport and allocation of sugars from source to sink cells (Kühn and Grof, 2010). Many sucrose transporters have been characterized in *Arabidopsis*, grape, and rice (Sauer and Stolz, 1994; Davies et al., 1999). Despite the progress made in identifying genes encoding sugar transporters, little is known about the transcriptional regulation of these genes. While some of the genes involved in sugar metabolism in kiwifruit have been identified (Nardozza et al., 2013), further studies on the regulation of sugar metabolism, especially in fruit tissue, are needed to develop approaches to regulate and improve fruit quality.

Genetic studies and variety breeding in kiwifruit are complex and time-consuming due to its high level of heterozygosity, and long juvenility period. High-throughput sequencing has now become a powerful tool for studying the transcriptome of species with and without sequenced genomes (Wang et al., 2013; Chen et al., 2014; Wu et al., 2014). Crowhurst et al. (2008) generated a collection of 132,577 expressed sequence tags (ESTs) in four *Actinidia* species (*A. chinensis, A. deliciosa, A. arguta, and A. eriantha*). Recent efforts using next-generation sequencing data have produced a draft genome sequence of a heterozygous kiwifruit “Hongyang.” In addition, an Illumina HiSeq 2000 sequencing platform has been used to generate transcriptomic data from three stages of fruit development in order to facilitate gene prediction and annotation (Huang et al., 2013). Recently, the transcript profiles of kiwifruit were constructed and analyzed by Li et al. (2015), with a focus on the secondary metabolism including phytohormones, sugars, starch, anthocyanin, and L-ascorbic acid.

At the RNA level, alternative splicing (AS) of pre-mRNAs represents a major mechanism by which the complexity of the transcriptome and proteome is increased (Modrek and Lee, 2002; McGuire et al., 2008; Bartlett et al., 2009; Tang et al., 2013), while long noncoding RNAs (lncRNAs) constitute a crucial regulatory module in diverse gene-silencing pathways (Bardou et al., 2014; Liu et al., 2015). Li et al. (2015) analyzed exon number, transcripts sizes, start sites of the novel transcripts and listed the extend 5′ or 3′ sites of alternative splicing events in different developmental stages of kiwifruit. However, the sequence and annotation of novel transcripts and other splicing events (exon skipping and intro retention) were not listed. In the present study, by employing sequence data derived from the nine different RNA-Seq libraries, we identified 7051 lncRNAs, 29,327 alternative splicing events and 2980 novel genes that were not annotated in the draft genome of “Hongyang” (Huang et al., 2013). Differential gene expression was also characterized between fruits at 20, 120, and 127 (7 days postharvest) days after pollination (DAP) to gain further insight into the genetic regulation of fruit development. Both up- and down-regulated genes were identified at each stage of fruit development. Since sugars are such an essential aspect of fruit flavor in kiwifruit, qRT-PCR was used to specifically study the expression level of genes putatively associated with sugar metabolism.

## Materials and methods

### Plant material

*A. chinensis* “Hongyang” was grown in the experimental station of Sichuan Academy of Natural Resources, Sichuan Province, China. Fruit samples were collected from five 5-year-old plant, at 20 days after pollination (DAP; beginning of cell division), 120 DAP (onset of fruit mature, Brix 6.5–7.5), and 127 DAP (onset of postharvest ripening, Brix 9-10). The sampled tissues were immediately frozen in liquid nitrogen and stored at −80°C for RNA-seq analysis. Two to three biological replicates were collected at each sampling. The physical or physiological parameters of tissues/organs of kiwifruit “Hongyang” were described in Huang et al. (2013).

### RNA extraction, transcriptome, and gene expression profile sequencing

Total fruit RNA was isolated using Trizol reagent, treated with DNase I and further purified with RNA clean kit (Promega, USA). RNA quality and quantity were checked with an Agilent 2100 Bioanalyzer RNA Nanochip (Agilent, Santa Clara, CA) and NanoDrop ND-1000 Spectrophotometer (NanoDrop, Wilmington, DE), respectively. RNA samples were pooled from equal amounts of RNA from five independent individuals. The samples were sent to Beijing Genomics Institute-Shenzhen (BGI, Shenzhen, China) for RNA-Seq library construction and sequencing using an Illumina HiSeq 2000 sequencing platform following manufacture's protocols (Illumina Inc, USA). Raw RNA-Seq reads have been deposited in the NCBI sequence read archive (SRA) under the accession number SRA065642 (Huang et al., 2013). In addition, the templates of RT-PCR or qRT-PCR were synthesized by using HiFiScript Reverse Transcription kit CW2582 (CWBIO, China), with oligo dT and random primers.

### RNA-seq read processing, assembly, and transcript construction

The adaptor and low quality bases were trimmed from raw sequencing reads using Trimmomatic (Bolger et al., 2014), and trimmed reads shorter than 40 bp were discarded. The resulting high quality reads were aligned to a ribosome RNA database (Quast et al., 2013) using Bowtie (Langmead et al., 2009) allowing up to three mismatches, and mapped reads were discarded in the subsequent analyses. The remaining cleaned reads were aligned to kiwifruit genome sequences using Tophat2 (Trapnell et al., 2009) allowing two mismatches for paired-end reads, and one mismatch for single-end reads. Only reads with perfect matches to the genome were used for reference-guided *de novo* assemblies using Cufflinks (Trapnell et al., 2010). The assembled transcripts from three different fruit developmental stages were merged together with a kiwifruit gene model using Cuffmerge, which was provided in the Cufflinks package.

### Identification of lncRNAs, novel genes, and as events

The assembled transcripts were translated into proteins using ESTScan (Iseli et al., 1999), and the longest protein for each transcript was kept and compared against the *Arabidopsis thaliana* protein and UniProt (TrEMBL and SwissProt) databases using the BLAST program with an *E*-value cutoff 1e-4. The blast results were used to assess the coding potential of each assembled transcript using Coding Potential Calculator (CPC; Kong et al., 2007).

To identify lncRNAs, transcripts originally obtained from the kiwifruit predicted gene models or those shorter than 200 bp were first discarded. The remaining transcripts with a CPC score < 0 and an ORF length < 300 bp were identified as lncRNAs. Novel protein-coding genes were identified using the following criteria: (1) they had to be located in intergenic regions and their distance to the closest predicted gene models should be >500 bp, (2) their ORF length should be longer than 300 bp, and (3) their CPC scores should be >0.

AS events were identified from the assembled transcripts using ASTALAVISTA (Foissac and Sammeth, 2007). Different categories of AS events were identified and counted using an in house Perl script (Sammeth et al., 2008).

### Differential gene expression

The number of clean reads that mapped to each kiwifruit gene model was calculated, and then normalized into fragments per kb exon model per million mapped fragments (FPKM). To identify differentially expressed genes during the fruit development, raw counts of RNA-seq expression data were first transformed using the get Variance Stabilized Data function in the DESeq package (Anders and Huber, 2010). The variance-stabilizing transformed expression data were then fed to the LIMMA package (Smyth, 2004), and *F*-tests were performed. Raw *P*-values were adjusted for multiple testing using the Benjamini-Hochberg procedure (Benjamini and Hochberg, 1995). Genes with a ratio between the maximum and minimum expression levels ≥2 and an adjusted *P* < 0.01 were identified as differentially expressed genes during fruit development.

### RT-PCR and qRT-PCR

Primers were designed to amplify lncRNAs, AS events and new genes. PrimeSTART HS DNA polymerase (Takara, China) was used for lncRNAs and the amplification of new genes. The real-time reverse transcription, quantitative PCR (qRT-PCR) was carried out in a total volume of 20 μl, containing 10 μl of SoFast EvaGreen (Bio-Rad, USA), 0.4 μM of each primer, 6 μl of 1:50 diluted cDNA and 3.2 μl ddH_2_O. Thermal cycling consisted of a hold at 95°C for 30 s, followed by 40 cycles of 95°C for 5 s, and 60°C for 20 s. After amplification, samples were kept at 95°C for 15 s and 60°C for 1 min. The temperature was then gradually raised, by 0.5°C every 10 s, to perform a melt-curve analysis. Each sample was amplified in triplicate, and all PCR reactions were performed on the StepOne Real-time PCR System (AB applied Biosystem, USA). The ΔΔCt method was employed with *Actin* (*Achn107181*) and *18S* rRNA (NCBI Accession: AB253775) as endogenous controls (Li et al., 2010). Primers used for RT-PCR and qRT-PCR were listed (Table S1).

### Amino acids, organic acids, and sugars analysis

Kiwifruit samples at three development stages (20, 120, and 127 DAP) were used for the analysis of amino acids, organic acids, and sugars. Each stage contains three biological replicates. The detection of amino acids was according to Javelle et al. (2003). Organic acids were prepared from three stages samples each containing five fruits. Fruits were put into a homogenizer for homogenizing, and then 40 mL ddH_2_O be added to 10 g of pulp at 4°C for 4 h. Twenty microliter filtering supernatant was used for liquid chromatograph (Model LC-6AD; Shimadzu, Tokyo, Japan) analysis. HPLC separations were performed using a Diamonsil C18 reverse-phase column (250 mm × 4.6 mm × 5 μm) purchased from USA. The mobile phases were used 10 mm H_2_SO_4_ (pH = 2.6), at a flow rate of 0.5 mL/min. The column temperature was set at 30°C. Sugars were detected by HPLC-ELSD, using an carbohydrate analysis column (3.9 × 300 mm, 10 μm) at 25°C. The mobile phase was composed of water and acetonitrile (23:77) with a flow rate of 1 mL/min (Nishiyama et al., 2008). Standard of Glucose, fructose, sucrose, citric acid, malic acid were purchased from Sigma (St. Louis, MO, USA).

## Results and discussion

### Sequencing and overview of the RNA-seq dataset

RNA-Seq libraries were constructed from three independent biological replicates of whole fruit tissues collected at 20, 120, and 127 DAP. A total of 81.1 million and 25.5 million reads were generated from paired-end (PE) and single-end (SE) libraries, respectively, using an Illumina HiSeq 2000 sequencing platform. Removal of low quality, adaptor sequence, and rRNA contaminated reads resulted in a final number of 66.2 million PE and 24.4 million SE reads, respectively, among which ~87.5% PE reads and ~90.6% SE reads were mapped to the kiwifruit genome (Table S2). Among the mapped reads, ~95% of PE and 89% of SE reads were uniquely aligned.

Approximately 79.1% PE, and 73.7% SE reads were mapped to the kiwifruit genome with perfect matches. These mapped reads were used for reference-guided *de novo* assembly and assembled into 56,313, 64,892, and 57,656 transcript isoforms in fruits at 20, 120, and 127 DAP, respectively. This corresponded to 49,766, 56,456, and 48,176 gene models, respectively. All of the assembled isoforms, together with the full length transcripts from kiwifruit draft genome (Huang et al., 2013), were merged to remove redundancies. This resulted in 54,425 gene models and 105,632 isoforms, among which 39,040 gene models were from kiwifruit predicted. There were 12,185 isoforms that were not expressed (FPKM ≈ 0) at any of the three fruit developmental stages. The majority of them (11,255; 92.4%) were from kiwifruit predicted gene models. The remaining 930 trace-expressed isoforms were not included in any further analyses. In summary, a comprehensive set of 104,702 transcripts, corresponding to 54,422 gene models, was obtained from the three stages of kiwifruit development.

### Kiwifruit lncRNAs

**Figure 1:**
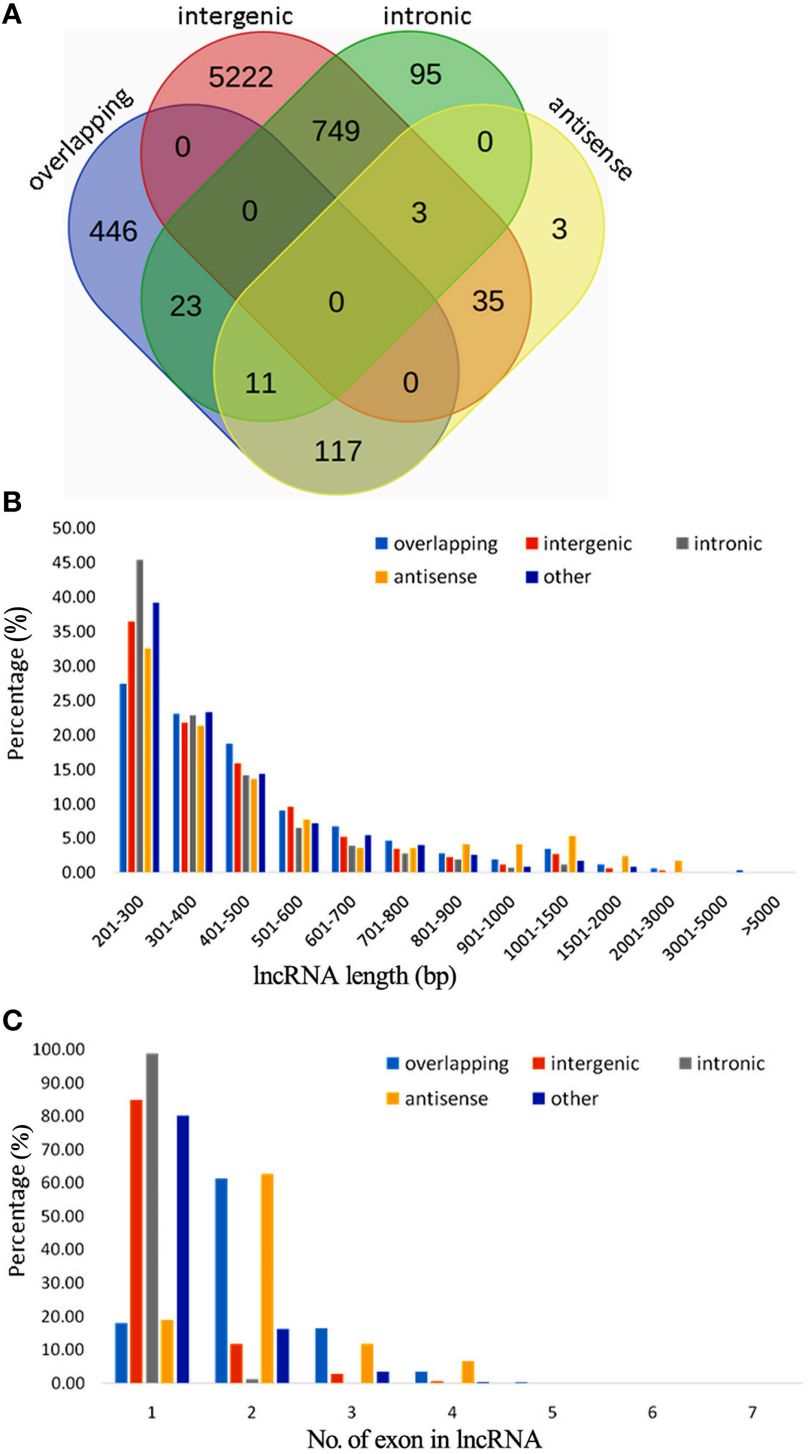
**Classification, length distribution, and exon number of lncRNAs in *A. chinensis* “Hongyang.” (A)** Venn diagram of lncRNAs categorized as overlapping, intergenic, intronic, and antisense lncRNAs; **(B)** Length distribution of lncRNAs, intronic, intergenic, antisense, and overlapping lncRNAs; **(C)** Number of exons in lncRNAs, intronic, intergenic, antisense, and overlapping lncRNAs.

A total of 7051 potential lncRNAs were identified in the comprehensive set of kiwifruit transcripts, among which 1511 had CPC scores between 0 and −1 and thus scored as “weak noncoding” while the other 5540 had CPC scores < −1 and thus scored as “strong noncoding” (Table S3A). The lncRNAs were placed into different groups based on the anatomical properties of their gene loci (Rinn and Chang, 2012). A subset of 6009 lncRNAs located in intergenic regions, with a distance >500 bp to the closest kiwifruit genes, were classified as intergenic large intervening noncoding RNAs (lincRNAs). Another 597 lncRNAs were categorized as overlapping lncRNAs since they were located in protein-coding gene regions. A total of 169 lncRNAs were categorized as antisense lncRNAs since they had more than a 50 bp overlap with their corresponding sense transcript. Finally, 881 were classified as intronic lncRNAs as they resided completely within an intron in protein-coding genes (Table 1). Importantly, this category system was not applicable to all the identified lncRNAs. A total of 347 lncRNAs were put into an “other group” since they did not confirm to any of the above categories. Additionally, in some cases, the same lncRNAs could be placed in different categories. As shown in the Venn diagram in Figure 1A, most of the antisense lncRNAs (117 out of 169) were also categorized as overlapping lncRNAs since they overlapped with kiwifruit protein-coding genes. Approximately 92% of the intronic lncRNA were also located within intergenic regions. Detailed information on each of the categorized lncRNAs including their genome location, transcript length, and expression profile during fruit development, are provided (Tables S3B–F).

**Table 1:** **Summary of lncRNAs and novel genes identified in the kiwifruit genome**.

**Features**	**No. of features**
**LncRNAs**	
IntergeniclncRNA (lincRNA)	6009
Overlapping lncRNA	597
IntroniclncRNA	881
IntroniclncRNA (pairs)	1736
Antisense lncRNA (lncNAT)	169
Antisense lncRNA (pair)	319
Noncoding-coding	302
Noncoding-noncoding	17
Other lncRNA	347
**NOVEL GENES**	
Transcript	4813
Gene	2980

A total of 6454 lncRNAs (~92%), were located in intergenic regions, including all 6009 lincRNAs (~85%) and other lncRNAs that partially resided in intergenic regions (Figure S1A). A similar proportion of intergenic lncRNAs (~93%) were identified in maize (Li et al., 2014), and 73% of lncRNAs were identified as lincRNAs in rice (Liu et al., 2012; Zhang et al., 2014). A total of 2910 (41.3%) kiwifruit lncRNAs overlapped with repeat sequences (Figure S1B; Table S3A). This proportion is close to that in rice (40%), but is lower than that in *Arabidopsis* (49%) and maize (68%; Liu et al., 2012; Li et al., 2014). The median length of kiwifruit lncRNAs is 364 nucleotides, which is between that of *Arabidopsis* and rice lncRNAs (Liu et al., 2012; Zhang et al., 2014). Antisense lncRNAs in kiwifruit tended to be longer than the lncRNAs in other categories, while intronic lncRNAs tended to have the shortest transcript length (Figure 1B). Consistent with maize, the majority of kiwifruit lncRNAs (83.4%) were single-exon transcripts. The exon numbers of lncRNAs in different categories exhibited significant variation. Approximately 80% of the antisense and overlapping lncRNA transcripts were multi-exon transcripts, while the intergenic and “other group” lncRNAs contained more than 80% single-exon transcripts; especially intronic lncRNAs, ~98.8% of which had one single exon (Figure 1C; Table 2).

**Table 2:** **Number of exons in different categories of lincRNAs in kiwifruit**.

**Exon number**	**No. of lncRNAs**	**No. of lincRNAs**	**No. of introniclncRNAs**	**No. of introniclncRNAs**
1	5581	5100	870	32
2	1134	709	11	106
3	279	169	0	20
4	47	25	0	11
5	7	5	0	0
6	2	1	0	0
7	1	0	0	0

The intronic and antisense lncRNAs appeared as pairs with their corresponding coding or noncoding transcripts. A total of 1736 and 319 of the intronic and antisense lncRNAs existed as transcript pairs, respectively (Tables S3G–I). A majority (94.7%) of the sense-antisense pairs consisted of one noncoding and one coding transcript, indicating a potential predominant cis-regulating role for these antisense lncRNAs (Table S3H). The other 17 were noncoding-noncoding pairs and further studies are needed to discern their function (Table S3I). Seven lncRNAs were randomly selected to verification by RT-PCR and Sanger sequencing, and six of them were found to be clearly expressed in the three fruit stages (Figure S2A). LncRNAs are thought to have a wide range of functions in the regulation of gene expression in higher plants (Bardou et al., 2014; Liu et al., 2015). Interestingly, three intronic lncRNAs were identified that overlapped with one neutral invertase (*Achn178991*) and two sugar transporter (*Achn017471* and *Achn319221*) genes involved in sugar and organic acid metabolism (Table S3G).

### Novel protein-coding genes

**Figure 2:**
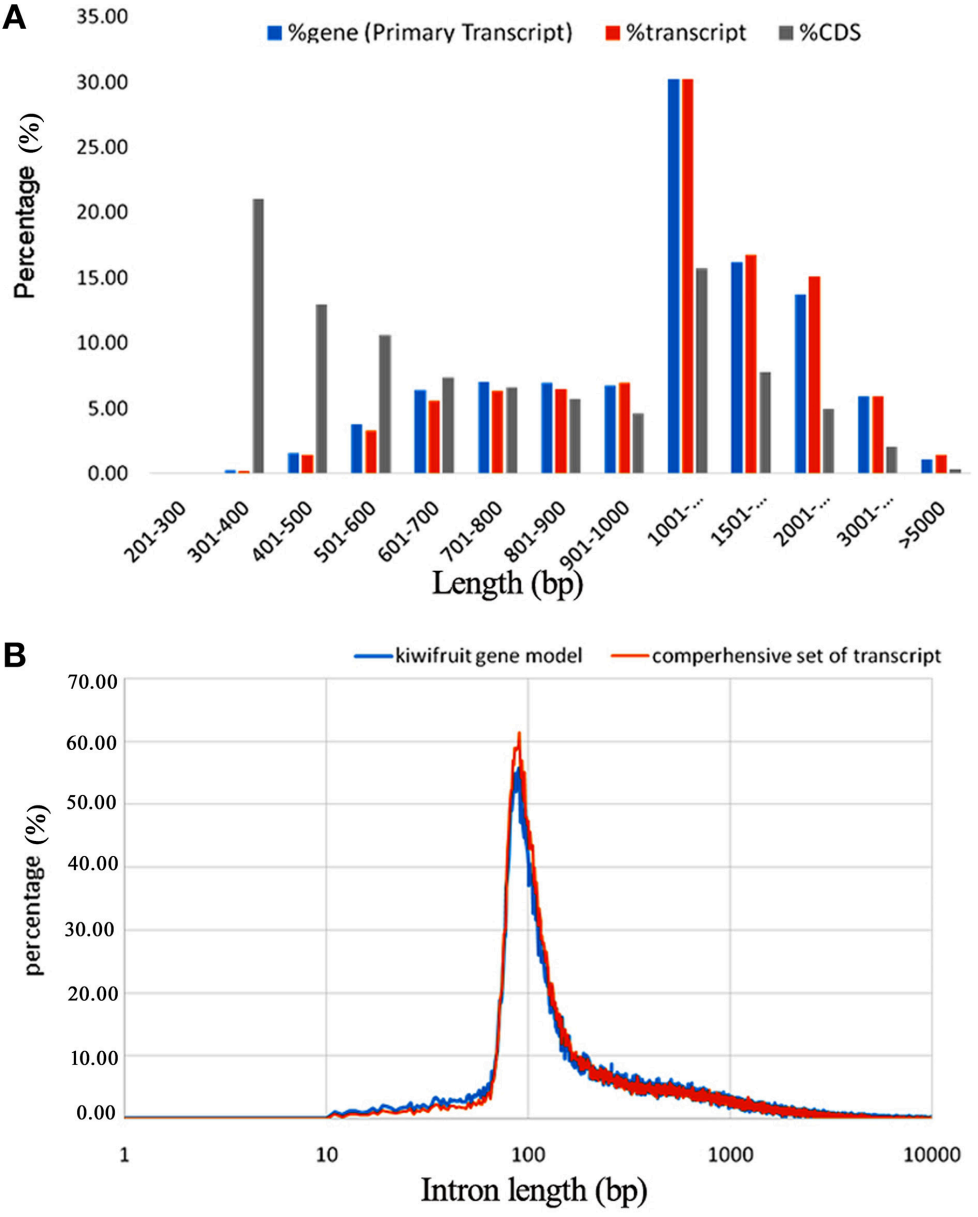
**Characteristics of newly identified protein-coding genes in the genome of *A. chinensis* “Hongyang.” (A)** Sequence length distribution of newly identified protein-coding genes; **(B)** Length distribution of introns in kiwifruit predicted genes (blue) and assembled transcripts (orange).

A total of 2980 novel potential protein-coding genes, containing a total of 4813 transcript isoforms, were identified in this study of kiwifruit (Table S4A). These protein-coding genes tended to have more exons and longer sequences than the identified lncRNAs. Nearly 84% (2498) of these novel genes were transcribed with multi-exons and more than 67% had primary transcript sequences longer than 1000 bp, and CDS sequences of ~30% of these novel genes were longer than 1000 bp (Figure 2A). Approximately 89% of the transcripts could be functionally annotated, indicating that the sequences obtained for these novel protein-coding genes were of high quality.

Four of the new genes, *MYB domain protein* (*Myb17*), *Flavanone 3-hydroxylase* (*F3H*), *F-box family protein* (*F-box*), and *SQUAMOSA promoter-binding-like* (*SPL*), which were randomly selected, were expressed in all three of the examined fruit stages. Sanger sequencing of the expressed genes were the same as the predicted sequences (Figure S2B). Interestingly, genes coding for ascorbic acid biosynthetic enzymes, including L-ascorbate oxidase (AO) and pectinesterase (PME), as well as glutamyl-tRNA reductase (GluTR) genes related to chlorophyll synthesis, and genes coding for anthocyanin biosynthesis, including chalcone isomerase (CHI), flavanone 3-hydroxylase (F3H) were identified among the novel genes (Table S4B).

### Alternative splicing (AS) events

By removing 11,255 non-expressed isoforms from the kiwifruit predicted genes, a total of 29,327 AS events were identified from transcript isoforms that were all expressed during kiwifruit fruit development (Table 3). These AS events occurred in 11,868 intron-containing genes, and represented 40,109 different transcripts (Table S5A). These data indicate that 26% of the expressed gene loci (36.7% of intron-containing gene loci), or 42.9% of expressed transcripts, were subject to alternative splicing. These results are comparable to the level of alternative splicing reported in other plant species (Filichkin et al., 2010; Zhang G. et al., 2010; Marquez et al., 2012; Shen et al., 2014; Thatcher et al., 2014). Among the AS events, 6930 belonged to the category of alternative 3′ acceptor site, 3118 to the alternative 5′ donor site category, 5414 to intron retention category (Table 3). The result is consistent with the previous study (Li et al., 2015). While, the total number of exon skipping category (5960) is more than Li et al. reported (2015). Interestingly, in contrast to the AS categories reported in other plant species (Reddy et al., 2013), the intron retention category was not the predominant AS event type in kiwifruit. Identification of AS events was confirmed using individual RNA-Seq data at various stages in different biological replicates. These data confirmed that intron retention was still not the predominant category of AS (data not shown). The percentage of different AS types, especially intron retention events, is likely highly correlated with intron length (Reddy, 2007). Most introns in kiwifruit were ~100 bp in length (Figure 2B). However, the average intron lengths in predicted kiwifruit genes and transcript isoforms were 1188 and 1082 bp, respectively, which were much larger than the average intron length in *Arabidopsis* (~170 bp) and rice (~430 bp; Reddy, 2007). Therefore, the lower percentage of intron retention events in kiwifruit may be due to the longer intron length. Interestingly, dramatic changes in the number of AS events in different fruit developmental stages were also observed. In general, the number of AS events during fruit development increased from 20 to 127 DAP. At 20 DAP and 120 DAP, intron retention was the second most abundant type of AS event, representing 17.5 and 21% of the total AS events, respectively. Although the percentage of intron retention events was decreased slightly from 21% at 120 DAP to 17.4% at 127 DAP, the percentage of exon skipping events increased greatly from 11.8 to 24.5%, which was accompanied by a concomitant decrease in the percentage of alternative 3′ acceptor and alternative 5′ donor AS events (Table 4). Changes in the percentage of AS types may be related to the physiological and biochemical changes that occur during fruit ripening. GT-AG represented ~98.3% of the splicing sites in the 207,186 introns of the expressed isoforms. The number of GC-AG, AT-AC, and others splicing sites was 2322 (1.1%), 403 (0.2%), and 740 (0.4%), respectively (Table 3). These numbers and percentages are consistent with those found in other plant species (Reddy et al., 2013). Five of the AS events, Auxin-response factor gene *Achn271111* (*TCONS_00051294, TCONS_00051295, TCONS_000512947*); b:UDP-glycosyltransferase gene *Achn017071* (*TCONS_00020411, TCONS_00020412*), were randomly selected to verification by RT-PCR and Sanger sequencing (Figure S3).

**Table 3:** **Number and percentage of different types of AS events and splicing sites**.

**AS events and slicing site**	**Number**	**Percentage (%)**
**AS EVENTS**		
Alternative 3′ Acceptor	6930	23.6
Alternative 5′ Donor	3118	10.6
Intron retention	5414	18.5
Exon skipping	5960	20.3
Others	7905	27.0
Total	29,327	100.0
**SPLICING SITE**		
GT-AG	203,721	98.3
GC-AG	2322	1.1
AT-AC	403	0.2
Others	740	0.4
Total	207,186	100.0

**Table 4:** **Number and percentage of different types of AS events at different fruit developmental stages defined as the number of days after pollination (DAP)**.

**Events**	**20 DAP**		**120 DAP**		**127 DAP**	
	**Number**	**Percentage**	**Number**	**Percentage**	**Number**	**Percentage**
Alternative 3′ Acceptor	1667	41.9	1962	41.3	1922	31.4
Alternative 5′ Donor	640	16.1	692	14.6	849	13.9
Intron retention	698	17.5	996	21.0	1063	17.4
Exon skipping	495	12.4	558	11.8	1498	24.5
Others	480	12.1	537	11.3	789	12.9
Total	3980	100.00	4745	100.00	6121	100.00

### AS events involved in vitamin C, carotenoid, chlorophyll, and flavonoid metabolic pathways and ethylene signaling

Kiwifruit is well-known for its high nutritional value due to its high content of ascorbic acid (vitamin C). A number of AS events were identified in various genes involved in ascorbic acid biosynthesis, including aldonolactonase (*Alase*), L-ascorbate peroxidase (*APX*), D-galacturonic acid reductase (*GalUR*), GDP-D-mannose-3,5-epimerase (*GME*), L-galactose-1-phosphate phosphatase (*GPP*), inositol-3-phosphate synthase (*IPS*), polygalacturonase (*PG*), glucose-6-phosphate isomerase (*PGI*), *PME*, mannose-6-phosphate isomerase (*PMI*), phosphomannomutase (*PMM*), and myo-inositol oxygenase (*MIOX*). AS events were also identified in genes responsible for ascorbic acid regeneration from its oxidized forms, including dehydroascorbate reductase (*DHAR*) and monohydroascorbate reductase (*MDHAR*; Table S5B). Interestingly, Laing et al. (2015) demonstrated that ascorbate concentration in *Arabidopsis* is determined via an alternatively spliced, upstream, open reading frame that represses the translation of a downstream *GGP* (GDP-l-galactose phosphorylase) gene under high ascorbate concentration.

AS events in genes involved in the biosynthesis and metabolism of carotenoids, chorophylls, and flavonoids were also identified. Genes associated with carotenoid biosynthesis, including non-heme hydroxylases (*CHY*), 7,9,7′,9′-tetra-cis-lycopene isomerase (*CrtISO*), P450 hydroxylases (*CYP*), phytoene desaturase (*PDS*), phytoene synthase (*PSY*), zeta-carotene desaturase (*ZDS*), and zeaxanthin epoxidase (*ZEP*) exhibited spliced variants in kiwifruit (Table S5C). In wild barley (*Hordeum chilense*)*, HcPsy1* has large number of transcripts originated by alternative splicing of, and the coexistence of functional and non-functional forms, to regulated *PSY* activity and carotenoid biosynthesis (Rodríguez-Suárez et al., 2011).

AS events were also found in genes in the chorophyll metabolic pathway, including chlorophyll a oxygenase (*CAO*), *GluTR*, chlorophyll b reductase (*CBR*), chlorophyll synthase (*CLS*), pheophorbide a oxygenase (*PAO*), pheophytin pheophorbide hydrolase (*PPH*), and chloroplast stay-green protein (*SGR*). Additionally, genes in the flavonoid biosynthesis pathway, including chalcone synthase (*CHS*), *F3H*, dihydroflavonol 4-reductase (*DFR*), anthocyanidin synthase (*ANS*), and anthocyanidin 3-O-glucosyltransferase (*UDP-glucose*) also exhibited AS events (Table S5C).

Ethylene plays a pivotal role in fruit ripening in climacteric fruits, including kiwifruit (MacDiarmid and Gardner, 1993). Therefore, AS events in genes involved in the ethylene signaling pathway were investigated. AS events were identified in kiwifruit genes homologous to 1-aminocyclopropane-1-carboxylate oxidase (*ACO*), 1-aminocyclopropane-1-carboxylate synthase (*ACS*), ethylene responsive protein kinase (*CTR*), ethylene signaling protein (*EIN2*), green ripe (*GR*), green ripe-like (GRL), and tetratricopeptide repeat protein (*TPR*; Table S5D). In pea (*Pisum sativum*), *PsACS1* has two transcripts originated by alternative splicing to response indole-3-acetic acid conditions that induce ethylene synthesis (Peck and Kende, 1998).

### Differential gene expression during fruit development

To analyze genes related to fruit development, gene expression levels were estimated by counting the number of aligned reads to each kiwifruit gene region and converting them to FPKM values (Table S6A). While high correlation coefficients among biological replicates (>0.85) were observed in general, one of the 20 DAP biological replicates was not included in the differential expression analysis due to a low correlation coefficient with the other two biological replicates (Table S7). Significant differences in both up- and down-regulated genes were identified in each stage of fruit development by comparing expression levels with the previous time point (Figure S4; Table S6B).

**Figure 3:**
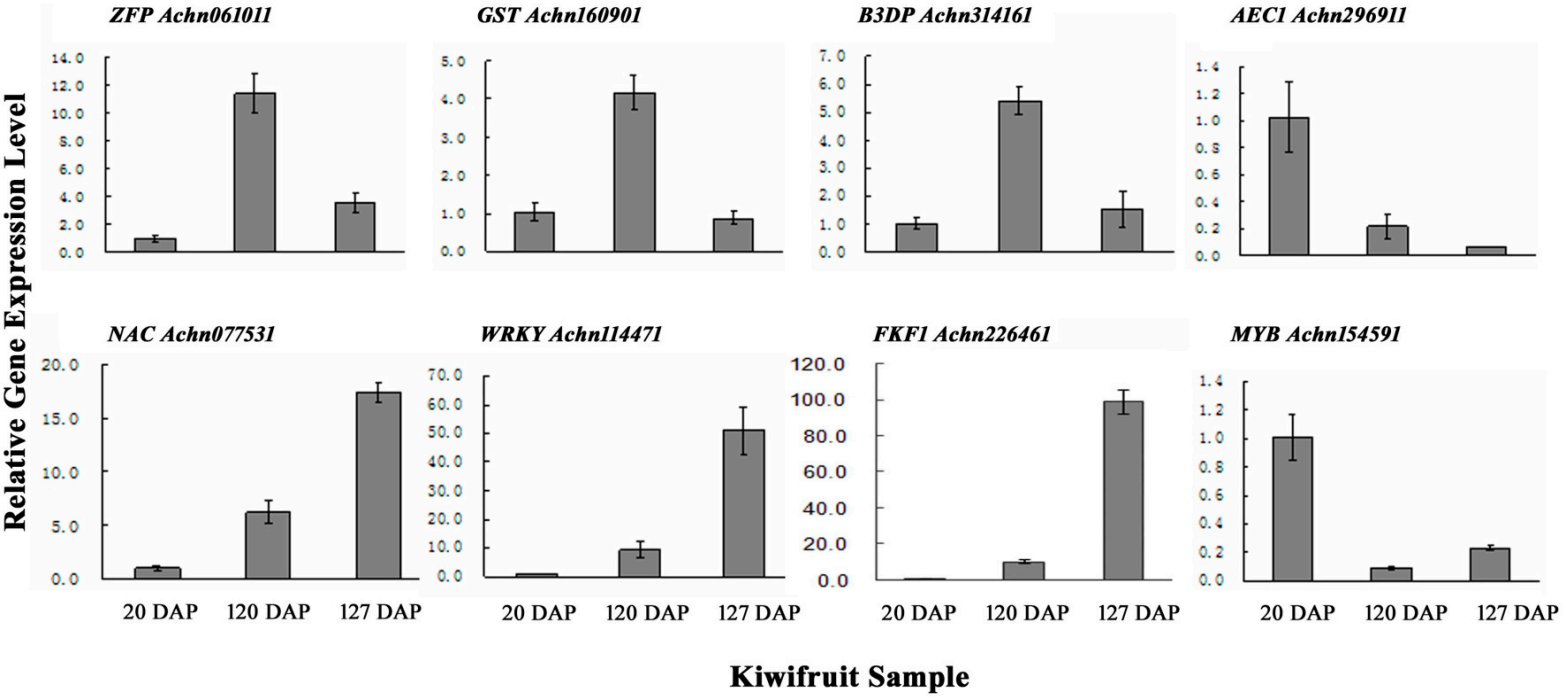
**qRT-PCR validation of genes randomly selected from gene expression profiles**. *ZFP*, Zinc finger protein; *GST*, glutathione s-transferase; *B3D*, B3 domain containing transcription repressor; *AEC*, auxin efflux carrier; *NAC*, NAC domain-containing protein; *WRKY*, WRKY transcription factor; *FKF1*, circadian clock-associated FKF1; *MYB*, MYB transcription factor. Error bars are standard error (SE) of three replicates.

To verify the results obtained from the RNA-Seq data, qRT-PCR was performed on a subset of 10 genes using gene-specific primers (Table S1). The 10 transcripts were selected due to their significantly different levels of expression at 20, 120, and 127 DAP as determined by their FPKM values. The results of the qRT-PCR indicated that eight of the ten selected genes exhibited expression levels similar to the levels indicated by their FPKM values (Figure 3; Table S6C).

A total of 5931 differentially expressed genes were identified during fruit development in kiwifruit. More specifically, 4395 genes were differentially expressed in fruits at 20 DAP and 120 DAP; 1739 of which were up-regulated and 2657 of which were down-regulated in fruits at 120 DAP compared to fruits at 20 DAP (Table S6A). Only two, *1-aminocyclopropane-1-carboxylate oxidase* (*ACC, Achn213921*) and *kiwellin* (*Achn022471*), of the 10 most highly up-regulated genes have been previously characterized. ACC plays an important role in the biosynthesis of the plant hormone ethylene, which in turn plays a major role in regulating fruit ripening (MacDiarmid and Gardner, 1993). Kiwellin, an allergenic protein formerly isolated from green kiwifruit, exhibits IgE binding capacity (Tuppo et al., 2008). Eighteen of the combined top 10 up- and top 10 down- regulated genes had unclear functions. A seed maturation protein gene (*Achn286381*) was highly expressed in mature fruit (120 DAP), while two *aspartic proteinase nepenthesin* genes (*Achn141951* and *Achn146881*) exhibited lower expression levels in mature fruit compared to immature fruit (Table S6D).

A comparison of fruits at 127 and 120 DAP also revealed significant variations in expression. A total of 4091 genes, comprised of 1670 up-regulated and 2421 down-regulated genes, were identified in fruits at 127 DAP. The 10 most up-regulated and 10 most down-regulated genes are listed (Table S6D). Three amylase genes (*Achn001191, Achn322221*, and *Achn141771*) were among the top 10 most highly up-regulated genes at the postharvest stage (127 DAP) of fruit development. The up-regulation of amylase genes is consistent with the results reported by Richardson et al. (2011). Limited information is available for 17 of the 20 most highly differentially expressed genes (Table S6D).

### Candidate genes related to kiwifruit sugar and organic acid metabolism

**Figure 4:**
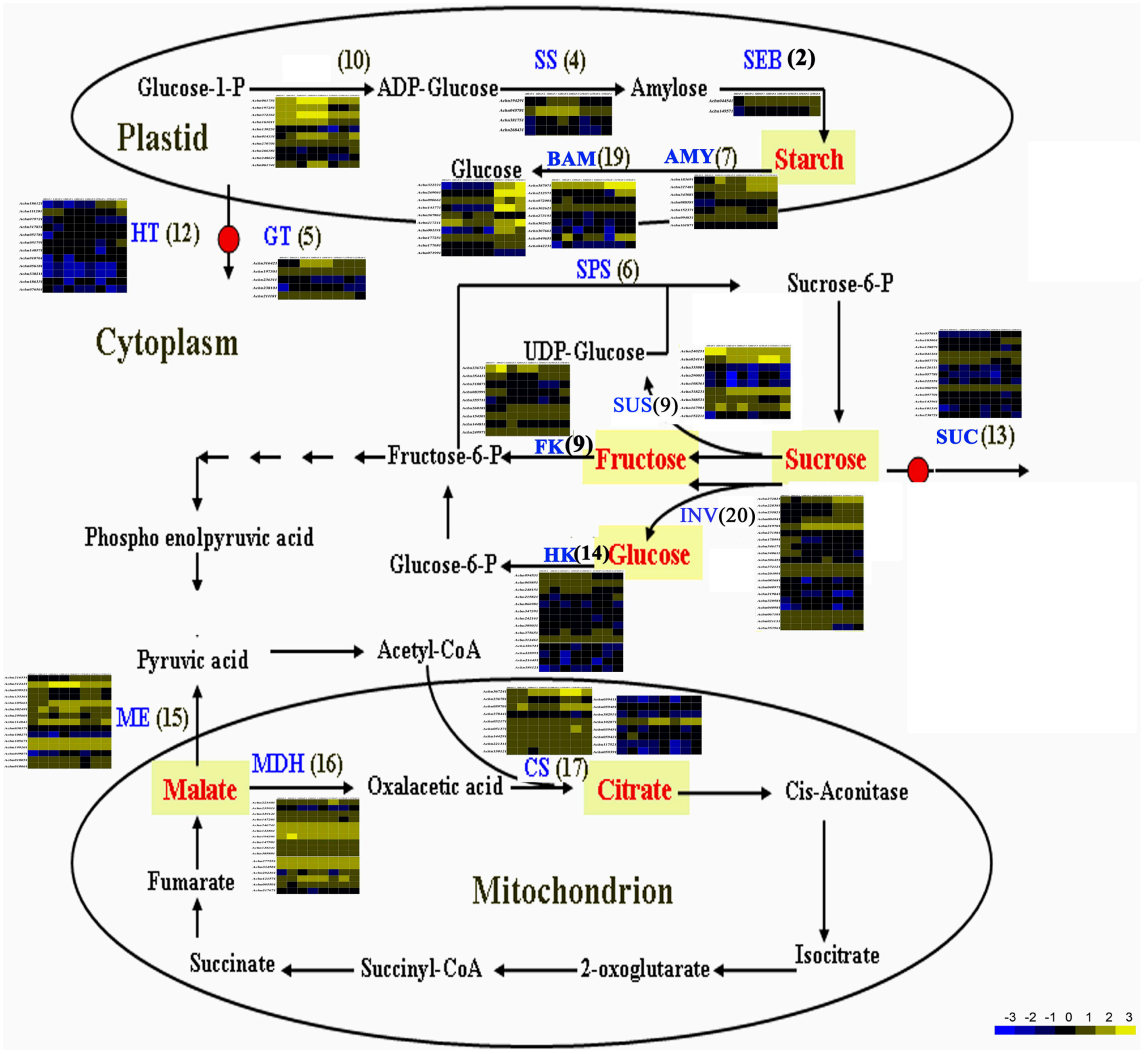
**Expression pattern of genes involved in sugar and main organic acid biosynthesis pathways in *A. chinensis* “Hongyang.”** The schematic diagram was illustrated according to Deluc et al. (2007) and Yin et al. (2010), with some modifications. AGPaseglucose-1-phosphate adenylyltransferase; AMY, α-amylase; BAM, β-amylase; CS, citrate synthase; FK, fructokinase; GT, glucose transporter; HK, hexokinase; HT, Hexose transporter; INV, invertase; ME, malic enzyme; MDH, malate dehydrogenase; SEB, starch branching enzyme; SPS, sucrose phosphate synthase; SS, starch synthase; SUS, sucrose synthase; SUC, sucrose transporter.

**Figure 5:**
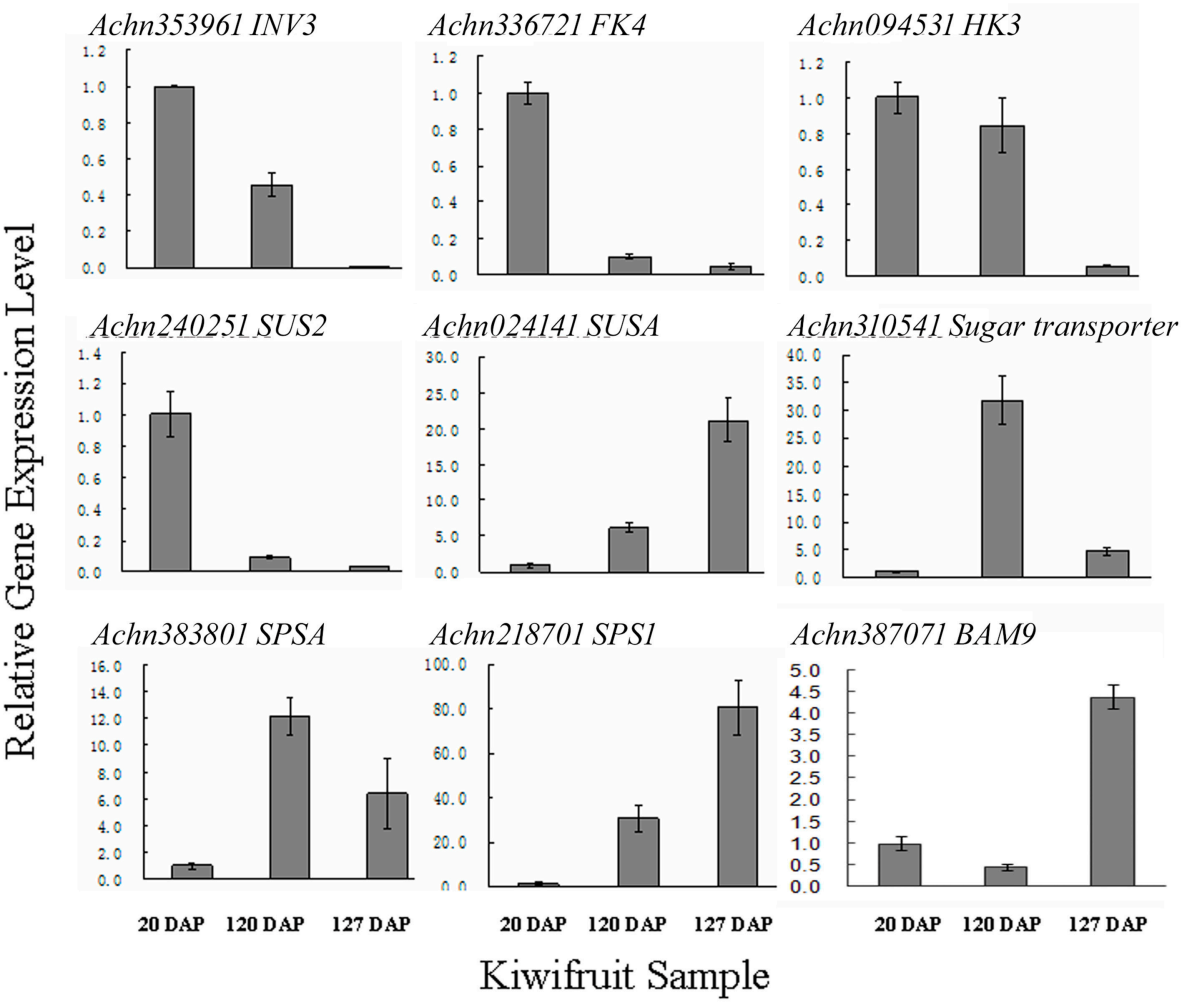
**qRT-PCR validation of RNA-seq expression data of sugar metabolism genes in *A. chinensis* “Hongyang.”** *INK3*, invertase 3; *FK4*, fructokinase; *HK3*, hexokinase 3; *SUS2*, sucrose synthase 2; *SUSA*, sucrose synthase A; *SPSA*, sucrose-phosphate synthase; *SPS1*, sucrose phosphate synthase 1; *BAM9*, β-amylase 9. Error bars are SE of three replicates.

The flavor of kiwifruit is highly dependent on the balance between soluble sugars and non-volatile organic acids (Nishiyama et al., 2008). Sweetness is the most important quality trait for kiwifruit as it influences overall fruit flavor (sugar/acid balance, perception of volatiles), and determines consumer acceptability (Nardozza et al., 2013). Most breeding programmes, however, have a negative impact on this trait. “Hongyang,” in comparison to other *A. chinensis* varieties, can accumulate more sugar levels (Wang et al., 2003; Nishiyama et al., 2008). Therefore, the expression of genes putatively related to kiwifruit sugar synthesis and accumulation and organic acid metabolism were analyzed (Figure 4; Table S8). Ten AGPase (Glucose-1-phosphate adenylyltransferase) genes, which play a key role in regulating starch biosynthesis, were identified in kiwifruit, and four of them were differentially expressed in different stages of fruit development. Four starch synthase (*SS*) genes were expressed in all three stages of fruit development, and two of them were more highly expressed in mature (120 DAP) fruit. The expression of two starch branching enzyme genes (*SBE*) was also detected in all three stages of fruit development, but no significant differences were observed in their expression in any of the stages of fruit maturity. Nineteen β-amylase transcripts were detected, 10 of which exhibited significant differences in expression. Seven β-amylase genes had the highest level of expression in the postharvest stage (127 DAP) of fruit maturity. The qRT-PCR data also indicated that *BAM9* (*Achn387071*) exhibited the highest level in mature (127 DAP) fruit (Figure 5). Starch content is relatively higher in mature fruit and then begins to decrease during postharvest ripening. The content of glucose increased from 0.40 to 6.42 g/100 g fresh weight in three development stages (Table S9). Because of starch degradation, the amount of glucose, fructose and sucrose was higher in the postharvest stage.

Invertase (INV) is an enzyme that catalyzes the hydrolysis (breakdown) of sucrose. Twenty expressed invertase genes were identified in fruit tissues of kiwifruit, five of which exhibited differential expression. In rice, the gene vacuolar invertase 3 (*OsINV3*) related to endospermal starch accumulation by regulated the ratio of hexose/sucrose. The coordinated expression of *OsINV3* is considered to play an important role in acquiring sink strength for the start of elongation in all types of caryopses (Ishimaru et al., 2005). In kiwifruit, the *INV3* homolog (*Achn353961*) is highly expressed in immature fruit (20 DAP) and may be related to fruit starch accumulation (Figure 5; Table S8). *LIN5* is an invertase that plays a key role in regulating sugar content, fruit development, fertility, and hormonal levels in tomato (Fridman et al., 2004). In kiwifruit, the *Lin5-like* homolog (*Achn120291*) may also play a key role in regulating soluble sugar levels (Table S8). Sucrose synthase (SUS) catalyzes the reversible conversion of sucrose and a nucleoside diphosphate into nucleoside diphosphate-glucose and fructose. SUS is involved in the synthesis of UDP-glucose and ADP-glucose in *Arabidopsis*, which are compounds that are linked to cellulose and starch biosynthesis, respectively (Barratt et al., 2009). A total of nine *SUS* genes were found to be expressed in fruit tissues at all stages of development, two of which, *SUS2* (*Achn240251*) and *SUSA* (*Achn024141*), exhibited differential expression. *SUS2* was highly expressed in immature fruit tissues and gradually decreased in subsequent stages of fruit development. In contrast, the expression of *SUSA* gradually increased as fruit developed from immature to the postharvest ripening stage (Figure 5; Table 5).

**Table 5:** **Levels of expression of genes related to sugar and main organic acid metabolism at three different states of fruit development defined as days after pollination (DAP)**.

**Gene**	**Annotation**	**20 DAP**	**120 DAP**	**127 DAP**	**FDR**	**Max/Min**
Achn343081	Alpha-amylase	2.58	14.48	26.3	0.006664	10.19
Achn183691	Alpha-amylase (AYM3)	2.64	44.96	96.61	0.000097	36.59
Achn227481	Alpha-amylase (AYM1)	8.73	31.64	67.62	0.000378	7.75
Achn061751	AGPase	68.88	379.56	110.6	0.000491	5.51
Achn197251	AGPase	98.62	54.07	4.72	0.001301	22.02
Achn372361	AGPase (APL4)	178.97	565.99	219.81	0.003030	3.16
Achn161011	AGPase (APS1)	67.51	113.91	27.98	0.008322	4.07
Achn322221	Beta-amylase 3 (BAM3)	0.16	0.34	279.29	0.000042	1745.56
Achn269061	Beta-amylase	3.74	2.66	500.67	0.000072	188.22
Achn090661	Beta-amylase	11.18	11.49	62.96	0.000365	5.63
Achn141771	Beta-amylase	1.23	0.25	456.94	0.000806	1827.76
Achn367861	Beta-amylase	22.62	7.74	3.32	0.001060	7.95
Achn217211	Beta-amylase (BAM1)	56.64	38.77	232.06	0.001982	5.99
Achn001191	Beta-amylase	0.14	0.11	63.98	0.001985	581.64
Achn177251	Beta-amylase	28.08	22.51	8.92	0.002340	3.52
Achn177681	Beta-amylase	2.95	2.39	8.69	0.004716	3.64
Achn387071	Beta-amylase 9 (BAM9)	322.22	135.27	892.92	0.005978	6.6
Achn367241	Citrate synthase	14.75	70.07	858.12	0.001555	58.18
Achn236781	Citrate synthase	15.74	1.5	10.4	0.001859	12.52
Achn059701	Citrate synthase	49.82	33.3	88.59	0.004314	2.66
Achn336721	fructokinase 4 (FK4)	308.35	48.98	17.11	0.000608	20.67
Achn316421	Glucose transporter	4.02	65.09	20.27	0.000100	16.19
Achn094531	Hexokinase3 (HK3)	30.93	18.52	4.28	0.004447	6.08
Achn186121	Hexose transporter	0.23	0.17	29.92	0.000266	176
Achn111201	Hexose transporter	12.04	1.82	6.01	0.000878	7.43
Achn272821	Neutral invertase	7.31	7.78	61.78	0.000197	8.45
Achn228381	Neutral invertase	1.64	2.37	9.73	0.000330	5.93
Achn235821	Neutral invertase	2.03	2.33	8.69	0.000708	4.28
Achn004941	Neutral invertase	4.5	13.48	9.83	0.005495	3
Achn353961	Neutral invertase (INV3)	28.23	9.11	0.61	0.000389	44.05
Achn216531	Malic enzyme	2.71	6.6	11.53	0.002824	4.25
Achn312431	Malic enzyme	157.08	525.98	151.56	0.004608	3.47
Achn039921	Malic enzyme	11.21	1.84	10.51	0.006924	7.23
Achn133361	Malic enzyme	11.21	1.84	10.51	0.006924	7.23
Achn105661	Malic enzyme	34.74	98.25	69.49	0.007258	2.83
Achn221601	Malate dehydrogenase	23.66	16.3	39.53	0.007967	2.43
Achn383801	Sucrose phosphate synthase (SPSA)	10.8	51.56	172.74	0.000946	15.99
Achn218701	Sucrose-phosphate synthase 1 (SPS1)	6.62	34.54	58.67	0.001999	8.86
Achn240251	Sucrose synthase (SUS2)	955.58	136.5	107.14	0.000509	9.61
Achn024141	Sucrose synthase (SUSA)	25.29	90.83	424.19	0.002634	16.77
Achn183061	Sucrose transporter	5.2	3.49	9.62	0.006722	2.76
Achn041261	Sucrose transporter (SUC4)	34.9	19.16	10.36	0.008328	3.56
Achn117481	sugar transporter	10.29	1.33	0	0.000033	1148
Achn387141	Sugar transporter	46.3	3.22	5.54	0.000160	16.78
Achn146141	Sugar transporter	15.9	2.61	10.18	0.000487	6.91
Achn017471	Sugar transporter	5.67	44.09	60.13	0.000599	10.6
Achn310541	Sugar transporter	14.31	98.59	375.63	0.000635	26.25
Achn006691	Sugar transporter	4.44	2.83	50.24	0.000696	17.75
Achn151531	Sugar transporter	20.52	3.6	1.26	0.000838	16.32
Achn263241	Sugar transporter	42.35	0.3	3.47	0.001251	157.9
Achn013271	Sugar transporter	6.1	20.51	51.75	0.002113	8.48
Achn197531	Sugar transporter	5.52	23.96	10.59	0.002284	4.34
Achn174641	Sugar transporter	6.72	18	2.1	0.002573	8.57
Achn006061	Sugar transporter	2.42	2.6	32.41	0.003297	13.39
Achn338711	Sugar transporter	34.16	26.19	6.27	0.003724	6.54
Achn008891	Sugar transporter	17.58	6.21	10.99	0.003885	3.01
Achn093731	Sugar transporter	43.76	66.83	12.11	0.003932	5.52
Achn182691	Sugar transporter	21.79	1.6	0.53	0.004010	38
Achn254481	Sugar transporter	0.04	0.26	8.22	0.004338	205.5
Achn266661	Sugar transporter	11.51	12.95	3.66	0.006027	3.54
Achn014401	Sugar transporter	19.98	13.53	6.41	0.006067	3.73
Achn069411	Sugar transporter	5.09	21	26.06	0.006227	5.12
Achn146071	Sugar transporter	13.45	30.87	50.38	0.008718	3.75
Achn192731	Sugar transporter	27.71	74.93	41.55	0.009134	2.7
Achn129481	Sugar transporter	56.82	9.78	5.07	0.009175	9.1

Listed genes were those that were considered to be differentially expressed.

Fructose, one of the most important sugars in kiwifruit, can be phosphorylated to fructose-6-phosphate by fructokinases (FK; Granot, 2007). A total of nine FK genes were found to be expressed in kiwifruit, five of which exhibited different levels of expression during fruit development. qRT-PCR data indicated that *FK4* (*Achn336721*) was most highly expressed in immature (20 DAP) fruit (Figure 5). Hexokinase (HK) phosphorylates glucose, producing glucose-6-phosphate in most organisms. Expression of 14 different HK genes was detected in fruit tissues, but only *HK3* (*Achn094531*) exhibited differential expression. *HK3* was highly expressed in immature (20 DAP) and mature (120 DAP) fruits (Figure 5).

SPS is an important component of the plant sucrose biosynthesis pathway. Plant growth and productivity have been correlated with SPS activity in crop plants like maize and rice (Sharma et al., 2010; Okamura et al., 2011). SPS activity has also been correlated with sucrose accumulation in sugarcane stems (Grof et al., 2006). A total of six SPS genes were found to be expressed in kiwifruit, two of which exhibited higher levels of expression relative to the other *SPS* genes. qRT-PCR data indicated that *SPSA* (*Achn218701*) and *SPS1* (*Achn383801*) exhibit a low expression level in immature fruit, and a higher expression level in mature (*SPSA*) and postharvest ripening (*SPS1*) stages (Figure 5). The RNA-Seq data, however, differ somewhat from the qRT-PCR data for the *SPSA* gene (Table 5). The content of sucrose increased from 0.23 to 3.50 g/100 g fresh weight in three development stages (Table S9), which may be attributed to *SPSA* and *SPS1* expression.

Sucrose transporters play a central role, as they orchestrate sucrose allocation both intracellularly and at the whole plant level. Sugars are translocated in plants via sugar transporters, which are involved not only in long-distance sugar transport via the loading and unloading of phloem cells, but also in sugar allocation into source and sink cells (Anders and Huber, 2010; Kühn and Grof, 2010). A total of 13 sucrose transporter (*SUC*) genes were found to be expressed in fruit tissues of kiwifruit, two of which were expressed at a higher level relative to the other sugar transporter genes. A total of 61 sugar transporter, 12 hexose transporter (*HT*), and five glucose transporter (*GT*) genes were found to be expressed in the examined fruit tissues, 23, two, and one of which had higher levels of expression, respectively, relative to the other genes in the same families. The qRT-PCR data indicated that sugar transporter (*Achn310541*) exhibited its highest expression level in mature fruit and therefore may potentially play a role in starch accumulation. Sucrose metabolism, including the activity of sucrose synthases, SPSs, fructokinases, hexokinases, starch synthases, sucrose transporters, glucose transporters, hexose transporters, and UDP-galactose transporters, however, play a more essential role in sugar accumulation in kiwifruit.

Citrate and malate, products of the TCA cycle, are the main organic acids that, combined with sugars, play a key role in fruit flavor. The content of malic acid was 0.36, 2.31, and 2.38 g/100 g fresh weight, and the content of citric acid was 4.02, 9.33, and 9.74 g/100 g fresh weight, respectively, in three development stages (Table S9). Citrate synthase (CS) is the key enzyme in citrate biosynthesis. A total of 17 *CS* genes were found to be expressed in fruit, however only *Achn367241* showed a distinct pattern of expression (Figure 4). It was highly expressed in postharvest ripened fruit relative to the other *CS* genes. Genes encoding malic enzymes (ME), which synthesize pyruvate by decarboxylating malate, were also analyzed. A total of 15 ME genes were found to be expressed in fruit, and five of them were more highly expressed relative to the other *ME* genes. A total of 16 malate dehydrogenase genes were also found to be expressed in fruit, however only *Achn221601* showed a distinct pattern of expression (Figure 4).

### Candidate genes related to kiwifruit glutamine, aspartate, and arginine metabolism

The combination of sugars, organic acids and free amino acids results taste of fruit (Sorrequieta et al., 2010). We detected the content of 17 amino acids and γ-aminobutyric acid at three development stages (20, 120, and 127 DAP; Table S9). Glutamine, arginine, and aspartate are the major amino acids in kiwifruits, which is similar to the results reported by Redgwell and MacRae (1992). The content of arginine form 1.25 to 1.42 (mg/g Dry weight) is not significant difference in three development stages. Comparably, the content of aspartate (1.50, 0.86, 0.83 mg/g Dry weight) and glutamine (4.52, 1.20, 1.03 mg/g Dry weight) are decreased from 20 DAP to 127 DAP (Table S9). The key genes putatively related to kiwifruit arginine, aspartate, and glutamine metabolism were analyzed. Six glutamate synthase (*GOGAT*) genes, six glutamate dehydrogenase genes (*GDH*), and four glutamate decarboxylase (*GAD*) genes were expressed in all three stages of fruit development (Table S10). But one of *GDH* (*Achn063781*) was slightly higher expressed in immature (20 DAP) fruit. In *A. thaliana, AtGOGAT* T-DNA insertion line showed a reduction of glutamate and biomass under normal CO_2_ condition. According to the results, *AtGOGAT* played importance of ammonium assimilation in roots (Kojima et al., 2014).

GAD and GDH catalyze glutamate to γ-aminobutyric acid and α-ketoglutarate respectively, as metabolic nexuses to regulate carbon and nitrogen metabolism (Fait et al., 2011). In *Panax ginseng* C. A. Meyer, the expression of *PgGAD* gene was enhanced under various abiotic stresses (Lee et al., 2010). Three argininosuccinate synthase (*ASS*) genes and two argininosuccinate lyase (*ASL*) genes were expressed in among three stages of fruit development, but the *ASS* gene (*Achn280681*) was decreased in 127 DAP. In addition, the *ASL* gene (*Achn041381*) was highly expressed in mature fruit and postharvest stage (Table S10). In rice, normal root elongation requires arginine produced by *OsASS* and *OsASL* (Xia et al., 2014). Aspartate transaminase (AspAT) is an important enzyme in amino acid metabolism which catalyzes the interconversion of aspartate and α-ketoglutarate to oxaloacetate and glutamate (Brauc et al., 2011). Eleven *AspAT* genes were found and expressed in all three stages of fruit development (Table S10). In *A. thaliana*, overexpression the cytosolic *AtAspAT* influences amino acid metabolism and defense responses against *Botrytis cinerea* infection (Brauc et al., 2011). Those genes (*GAD, GDH, GOGAT, ASS, ASL, AspAT*) involved in glutamine, arginine, and aspartate metabolism may take part in stress response or development in kiwifruit.

## Conclusions

Kiwifruit is a highly heterozygous vine plant with limited genomic resources. “Hongyang,” the cultivar chosen for the current transcriptome study, is widely planted in China and represents an excellent reference for genetic studies in kiwifruit. The major achievement of the current study was, through sequence assembly, annotation, expression analysis, and identification and characterization of lncRNAs, AS events, and novel protein-coding genes, to provide a list of potential candidate genes that could serve as targets for genetic improvement of kiwifruit. The study also serves as a resource for the expression patterns of the candidate genes. Collectively, the data in this study represents a valuable resource for further studies of fruit development in kiwifruit.

## Author contributions

JL and YL conceived and planned the study; WT, YZ, MW, ZF, JL, and YL contributed to drafting the manuscript; WT, YZ, J. Dong, J. Yu, J. Yue, FL, XG, and SH performed the experiment and analyzed the data; JS, XN, and J. Ding collected the samples. The authors declare that they have no competing interests.

### Conflict of interest statement

The authors declare that the research was conducted in the absence of any commercial or financial relationships that could be construed as a potential conflict of interest.

## Supplementary material

Table S1 **Primers used for RT-PCR and qRT-PCR analysis**.

Table S2 **Summary statistics of RNA-Seq data**.

Table S3 **(A)** LncRNAs of *A. chinensis* “Hongyang”; (B) Overlapping lncRNAs; (C) Intergenic large intervening noncoding RNAs (lincRNAs); (D) Intronic lncRNAs; (E) Antisense lncRNAs; (F) Other lncRNAs; (G) Paired intronic lncRNAs; (H) Noncoding-coding pairs of antisense lncRNAs; (I) Noncoding-noncoding pairs of antisense lncRNAs.

Table S4 **(A)** Newly identified, potential protein-coding genes in the genome of *A. chinensis* cv. “Hongyang”; (B) Newly identified, novel genes involved in vitamin C, carotenoid, chlorophyll, and flavonoid metabolic pathways and ethylene signaling pathways.

Table S5 **(A)** Splice variants in *A. chinensis* cv. “Hongyang”; (B) Splice variants of vitamin C metabolic pathway genes; (C) Splice variants of chlorophyll, and flavonoid metabolic pathway genes; (D) Splice variants of ethylene signaling pathway genes.

Table S6 **(A)** Gene expression profiles in *A. chinensis* cv. “Hongyang” at three stages of fruit development and ripening; (B) Significantly differentially expressed genes of *A. chinensis* cv. “Hongyang” in three stages of fruit development and ripening; (C) FPKM values of genes randomly selected for realtime-PCR validation; (D) Top 10 differentially expressed genes in three stages of fruit development and ripening.

Table S7 **Correlation analyses of RNA-Seq replicates**.

Table S8 **Candidate genes involved in sugar and main organic acids biosynthesis in *A. chinensis* “Hongyang”**.

Table S9 **Physiological parameters of *A. chinensis* “Hongyang”**.

Table S10 **Candidate genes involved in amino acids biosynthesis**.

Figure S1 **(A)** Distribution of lncRNAs in gene region (orange) and intergenic region (blue) along the kiwifruit genome; **(B)** Distribution of lncRNAs overlapped with repeat region (orange) and non-repeat (blue) along the kiwifruit genome.

Figure S2 **Validation of lncRNAs (A) and newly identified genes (B) by RT-PCR and Sanger sequencing analysis**. *lncRNAs: TCONS_00088122, TCONS_00040823, TCONS_00084056, TCONS_00001512, TCONS_00077754, TCONS_00077829. Myb17: MYB domain protein 17; F3H: Flavanone 3-hydroxylase; F-box: F-box family protein; SPL: SQUAMOSA promoter-binding-like.*

Figure S3 **Validation of AS events by RT-PCR and Sanger sequencing analysis. (A)** Auxin-response factor gene *Achn271111* (*TCONS_00051294, TCONS_00051295, TCONS_000512947*); **(B)** UDP-glycosyltransferase gene *Achn017071* (*TCONS_00020411, TCONS_00020412*).

Figure S4 **Gene expression profiles during fruit development and ripening in *A. chinensis “*Hongyang.”** Two replicates of fruit samples at 20 DAP stage, and three replicates of fruit samples at 120 and 127 DAP stages were used to calculate expression levels.
