# Supplementary figures and images for: Comprehensive Transcriptome Profiling Reveals Long Noncoding RNA Expression and Alternative Splicing Regulation during Fruit Development and Ripening in Kiwifruit (Actinidia chinensis)
Source: Front Plant Sci. 2016 Mar 29;7:335. doi: 10.3389/fpls.2016.00335 (PMC5007456; doi:10.3389/fpls.2016.00335)

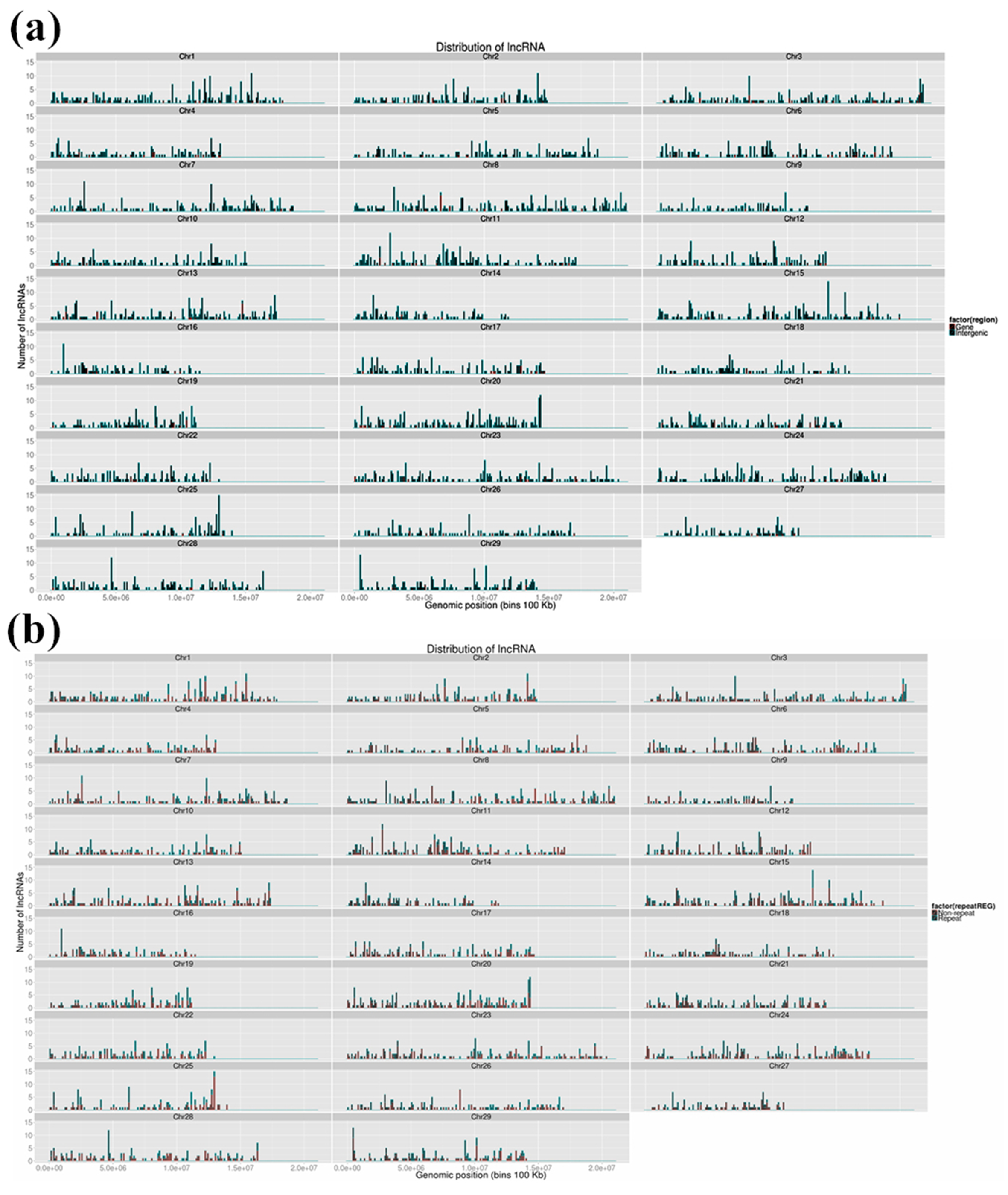

Supplement: Figure S1 — (A) Distribution of lncRNAs in gene region (orange) and intergenic region (blue) along the kiwifruit genome; (B) Distribution of lncRNAs overlapped with repeat region (orange) and non-repeat (blue) along the kiwifruit genome. [file Image1.TIF]

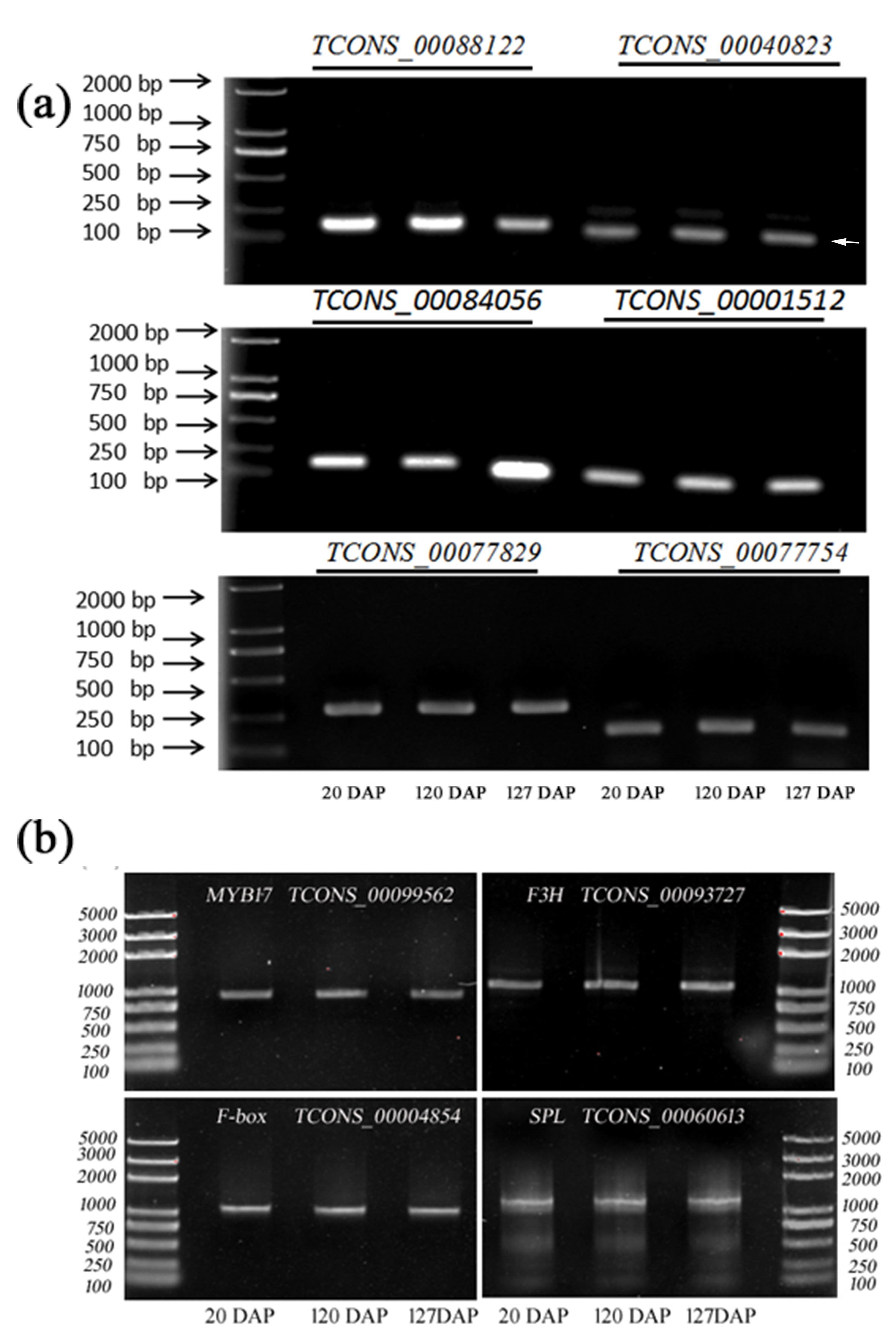

Supplement: Figure S2 — Validation of lncRNAs (A) and newly identified genes (B) by RT-PCR and Sanger sequencing analysis. lncRNAs: TCONS_00088122, TCONS_00040823, TCONS_00084056, TCONS_00001512, TCONS_00077754, TCONS_00077829. Myb17: MYB domain protein 17; F3H: Flavanone 3-hydroxylase; F-box: F-box family protein; SPL: SQUAMOSA promoter-binding-like. [file Image2.TIF]

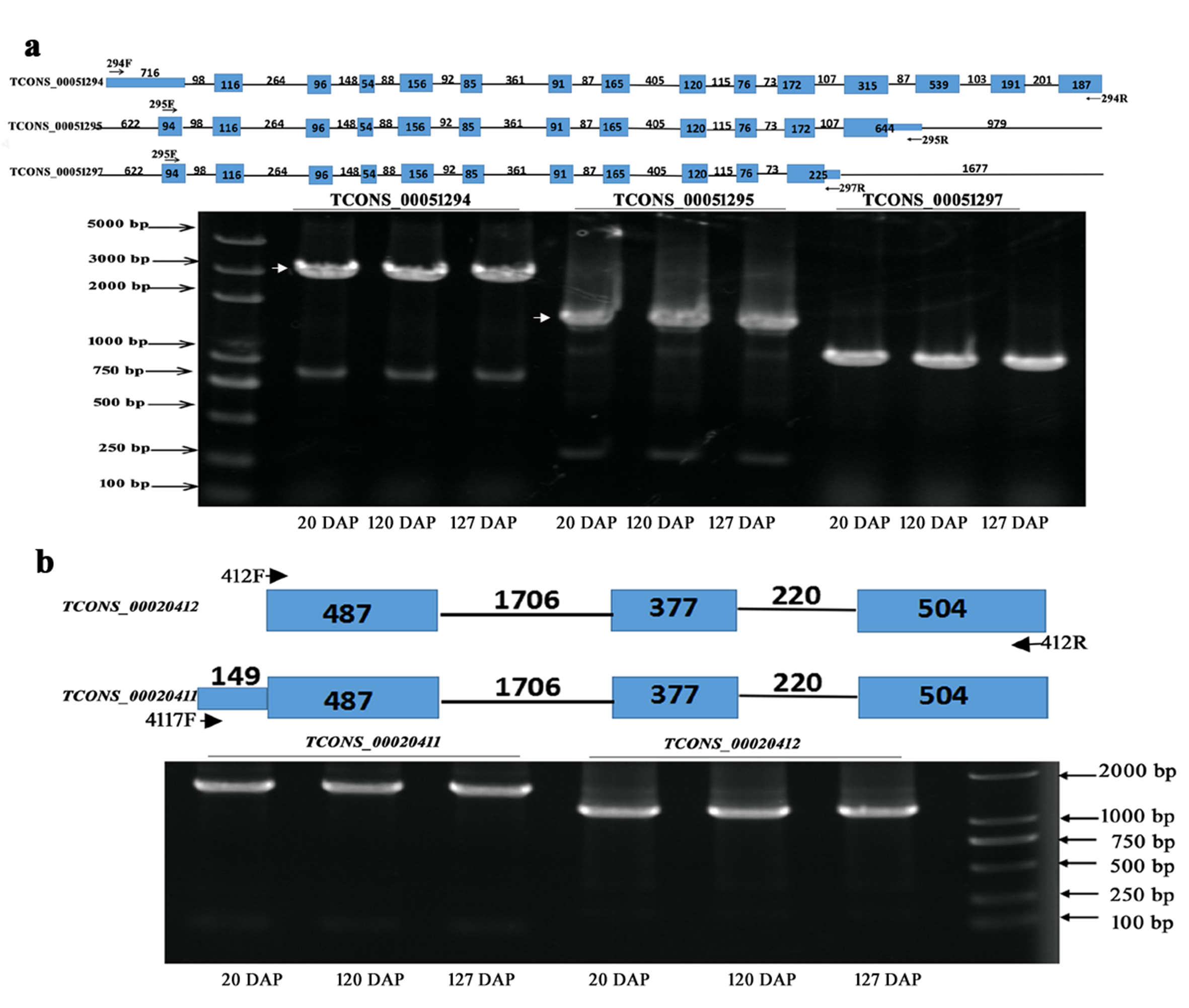

Supplement: Figure S3 — Validation of AS events by RT-PCR and Sanger sequencing analysis. (A) Auxin-response factor gene Achn271111 (TCONS_00051294, TCONS_00051295, TCONS_000512947); (B) UDP-glycosyltransferase gene Achn017071 (TCONS_00020411, TCONS_00020412). [file Image3.TIF]

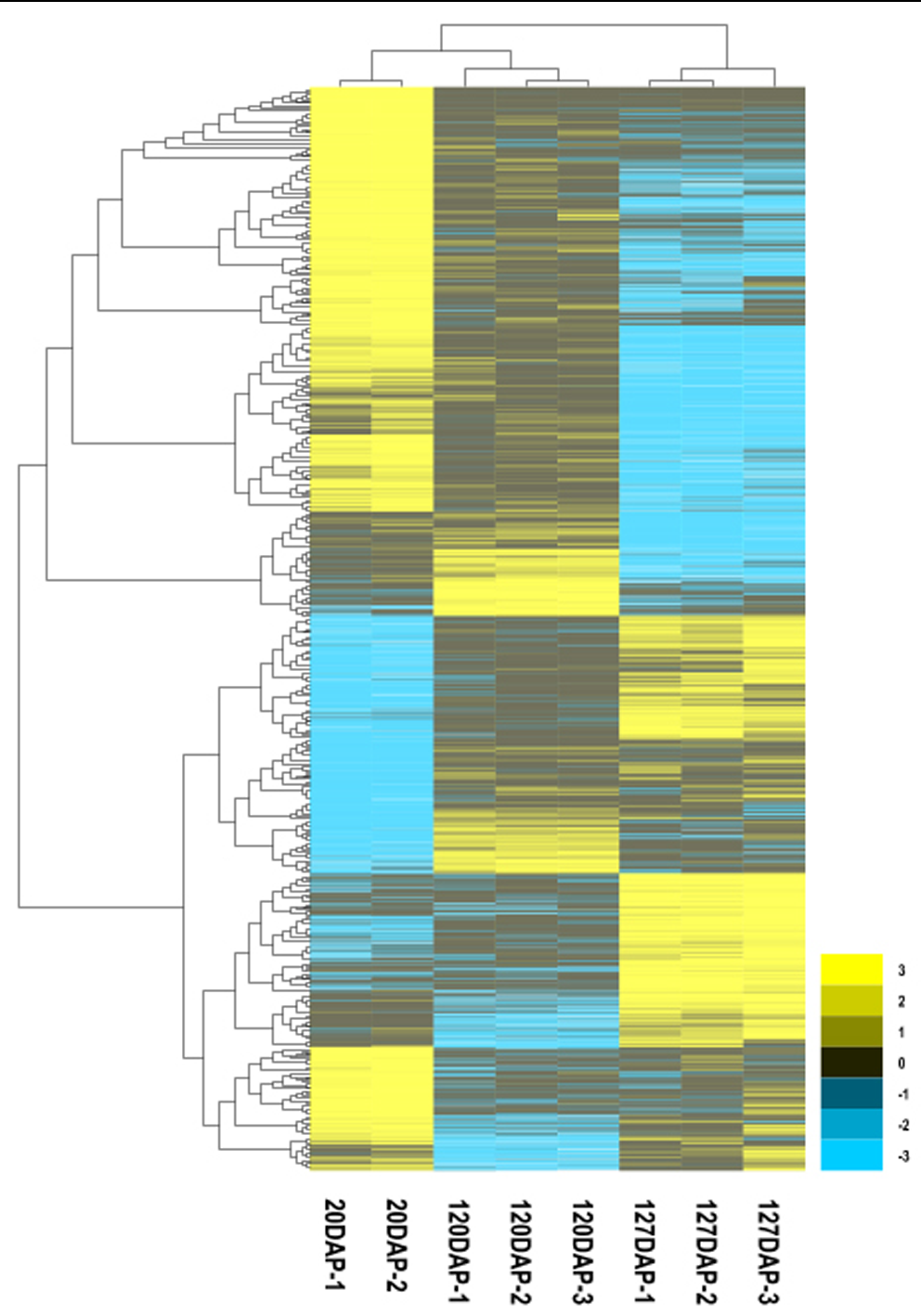

Supplement: Figure S4 — Gene expression profiles during fruit development and ripening in A. chinensis “Hongyang.” Two replicates of fruit samples at 20 DAP stage, and three replicates of fruit samples at 120 and 127 DAP stages were used to calculate expression levels. [file Image4.TIF]
